# Treatment of chronic active antibody-mediated rejection in renal transplant recipients – a single center retrospective study

**DOI:** 10.1186/s12882-019-1672-8

**Published:** 2020-01-06

**Authors:** Hsien-Fu Chiu, Mei-Chin Wen, Ming-Ju Wu, Cheng-Hsu Chen, Tung-Min Yu, Ya-Wen Chuang, Shih-Ting Huang, Shang-Feng Tsai, Ying-Chih Lo, Hao-Chung Ho, Kuo-Hsiung Shu

**Affiliations:** 1 Division of Nephrology, Department of Internal Medicine, Taichung Veterans General Hospital, Taichung, Taiwan; 2 Department of Pathology and Laboratory Medicine, Taichung Veterans General Hospital, Taichung, Taiwan; 3 Division of Urology, Department of Surgery, Taichung Veterans General Hospital, Taichung, Taiwan; 4 Division of Nephrology, Department of Internal Medicine, Lin Shin Hospital, No.36, Sec. 3, Hueijhong Rd., Nantun District, Taichung City, 40867 Taiwan

**Keywords:** Chronic active antibody mediated rejection, Kidney transplantation, Graft survival, Adverse events

## Abstract

**Background:**

Chronic active antibody-mediated rejection is a major etiology of graft loss in renal transplant recipients. However, there is no consensus on the optimal treatment strategies.

**Methods:**

Computerized records from Taichung Veterans General Hospital were collected to identify renal transplant biopsies performed in the past 7 years with a diagnosis of chronic active antibody-mediated rejection. The patients were divided into two groups according to treatment strategy: Group 1 received aggressive treatment (double filtration plasmapheresis and one of the followings: rituximab, intravenous immunoglobulin, antithymogycte globulin, bortezomib, or methylprednisolone pulse therapy); and group 2 received supportive treatment.

**Results:**

From February 2009 to December 2017, a total of 82 patients with biopsy-proven chronic antibody mediated rejection were identified. Kaplan-Meier analysis of death-censored graft survival showed a worse survival in group 2 (*P* = 0.015 by log-rank test). Adverse event-free survival was lower in group 1, whereas patient survival was not significantly different. Proteinuria and supportive treatment were independent risk factors for graft loss in multivariate analysis.

**Conclusions:**

Aggressive treatment was associated with better graft outcome. However, higher incidence of adverse events merit personalized treatment, especially for those with higher risk of infection. Appropriate prophylactic antibiotics are recommended for patients undergoing aggressive treatment.

## Background

Chronic active antibody mediated rejection (CAMR) is one of the most frequently encountered etiologies associated with graft failure. Diagnosis is based on the detection of donor-specific antibodies (DSAs) and specific morphologic lesions, most importantly, microvascular inflammation/injury with or without capillary C4d deposition [1]. A variety of treatment strategies have been proven effective for acute antibody mediated rejection [2]. On the contrary, treatment for CAMR has remained a major challenge.

Current therapeutic approaches for CAMR are based on retrospective studies and pilot trials, including intravenous immunoglobulin (IVIG) plus rituximab [3, 4], proteasome inhibitor-bortezomib [5], complement inhibitor-eculizumab [6], and IL-6 receptor blocker [7]. However, the results of these studies have not always turned out to be effective [4, 5], and the adverse events derived from these immunosuppressants are of great concern. Therefore, our study investigated the outcomes of CAMR in our center by comparing graft survival between different treatment strategies.

## Methods

### Patients and graft biopsies

Computerized records from Taichung Veterans General Hospital were collected to identify renal transplant biopsies performed in the past 7 years with a diagnosis of CAMR. The first biopsy was used for statistical analysis if the patient had multiple biopsies. All biopsies were performed for cause and reviewed by a renal pathologist. Biopsies with ABO-incompatable grafts and those with recurrent or de novo glomerulonephritis (GN) and DM nephropathy were excluded. All the patients had negative T and B cell complement-dependent cytotoxicity cross-match (CDC-CMX) result before kidney transplantation.

Thymoglobulin or basiliximab may be prescribed for induction therapy. Maintenance immunosuppression included calcineurin inhibitors (CNIs) tacrolimus or cyclosporine A, mycophenolate, and prednisone. mTOR inhibitor, either sirolimus or everolimus, was prescribed in few patients depending on the discretion of the physician.

One or more of the following treatment strategies were selected for CAMR treatment according to the patient’s clinical condition and decision of the individual practitioners: no treatment, methylprednisolone (MP) pulse therapy (usually 500 mg of MP for 3 days), double filtration plasmapheresis (DFPP), rituximab intravenous bolus (375 mg/m^2^), intravenous immunoglobulin (IVIG) (2 g/kg), and rabbit antithymocyte globulin (ATG) (Thymoglobulin 1–1.5 mg/kg for 3–5 days). DFPP was performed using Evaflux 4A as the plasma fractionator. The exchange volume was set at 1~1.5 times of plasma volume. Estimated plasma volume was 0.07 x weight (kg) x (1-hematocrit [Hct]). 300–500 mL saline solution was infused as the replacement fluid. In a few patients, bortezomib (1.3 mg/m^2^) was also used. Multiple treatments, usually yearly, were performed if follow-up graft biopsy revealed persistent lesions. The patients were divided into two groups according to treatment strategy. Group 1 received aggressive treatment (DFPP and one of the followings: rituximab, IVIG, ATG, bortezomib, or MP pulse therapy); and group 2 received supportive treatment. In group 1, patients were usually treated annually with DFPP plus one of the 5 drugs, but different in each year in order to accomplish a wide blockade of the alloimmunity. In group 2 (and also group 1), patients received routine medical care for chronic kidney disease, including ideal blood pressure control, blood sugar control, hyperuricemia control, and preventing further kidney damage by avoiding drugs such as nonsteroidal anti-inflammatory drugs (NSAIDs). Antihypertensive agents (ACEI or ARB), oral hypoglycemic agents or insulin, and urate-lowering therapy (allopurinol/febuxostat) were prescribed according to each patient’s clinical condition. Oral sodium bicarbonate was prescribed if the patient had metabolic acidosis (serum bicarbonate less than 22 mEq/L).

All of our colleagues are familiar with the care for chronic kidney disease patients. The control of lipids with statin/fibrate and blood pressure with ACEI/ARB are standard-of-care in our transplant team.

### End points

The patients were followed up until graft loss or death or the end of 2017. The definition of graft loss included: returned to dialysis, re-transplant, or patient death. Primary end point was graft survival after treatment in the 2 groups. Secondary outcome included patient survival and the occurrence of major adverse events. Major adverse event was defined by any event that was associated with death, admission to hospital, prolongation of a hospital stay, persistent or significant disability or incapacity, or was otherwise life-threatening in connection with specific treatment, according to the World Health Organization Good Clinical Practice guidelines.

### Histopathology and diagnosis of CAMR

All renal graft biopsies were performed using ultrasound-guided percutaneous technique (two~three cores per biopsy; 16~18 gauge needle). Graft biopsies were examined by light microscopy using silver methenamine and periodic acid-Schiff (PAS) stains, immunofluorescence studies for IgG, IgA, IgM, C3, C4d, C1q, kappa, and lambda light chains, and electron microscopy.

The same pathologist evaluated and graded graft biopsies according to Banff 2017 criteria [8]. Glomerulitis (g), peritubular capillaritis (ptc), transplant glomerulopathy (cg), interstitial fibrosis (ci), tubular atrophy (ct), and mesangial matrix (mm) scores were assigned in each case according to Banff parameters [1, 9]. C4d staining was performed on all biopsies by direct immunofluorescence on frozen sections.

For CAMR, all 3 criteria in the following were met for diagnosis according to Banff 2017 criteria: (1) morphologic evidence of chronic tissue injury, (2) evidence of current/recent antibody interaction with vascular endothelium, and (3) serologic evidence of donor-specific antibodies (DSA, to HLA or other antigens). C4d staining in the biopsy tissue or expression of validated transcripts/classifiers may substitute for DSA [8]. Determination of HLA antibody by Luminex® method is expensive in Taiwan and is not affordable to every patient. Gene expression tests are not performed routinely. For those who didn’t perform DSA, C4d staining should be positive for the definite diagnosis of CAMR.

### Data analysis

Normal distribution of the data was evaluated using the Kolmogorov-Smirnov test. Normally distributed data were expressed as mean ± standard deviations (SD), and non-normally distributed data as median and interquartile ranges (IQR). Categorical variables were shown as frequency (%). Fisher's exact test was used to compare categorical data, and the Mann–Whitney U test was used for comparison of continuous data. Kaplan–Meier analysis was applied for calculation of graft and patient survival or adverse events free survival. Log-rank test was used for comparison of survival between groups. To identify the predictors of graft loss in CAMR patients, we conducted univariate and multivariable analysis using the Cox proportional hazards regression model. A *P*-value of less than 0.05 was considered statistically significant. All statistical analyses were performed by using SPSS software (version 21.0, Chicago, IL, USA).

## Results

### Comparison of patient's demographics

From February 2009 to December 2017, a total of 85 patients with biopsy-proven CAMR were identified. Three cases were excluded from the analysis owing to short follow up duration (less than 6 months). Group 1 comprised 59 cases, whereas group 2 comprised 23 cases. In group 1, besides DFPP, 40 patients had received Rituximab, 10 patients had received IVIG, 10 patients had received bortezomib, whereas 4 patients had received antithymocyte globulin and 17 patients had received MP pulse therapy only.

There were no statistically significant differences between group 1 and group 2 in terms of age, donor type, transplant duration, follow up duration, percentages of diabetes mellitus, hepatitis B or C, panel reactive antibody (PRA) class I and II titer, percentages of patients who received induction treatment, immunosuppressive regimen (cyclosporine based or tacrolimus based), serum creatinine, proteinuria, and Banff scores (including cg, ci + ct, mm, g + ptc) (Table 1).

**Table 1:** Comparison of patient demographics between different treatment groups

	Group 1, Aggressive treatment (*N* = 59)	Group2, Supportive treatment (*N* = 23)	*P* value
Age at biopsy	50.5 [42.7–58.8]	55.6 [47.1–65.3]	0.052
Donor type			0.245
Deceased	43 (74.1%)	19 (86.4%)	
Living	15 (25.9%)	3 (13.6%)	
Transplant duration (mo)	95.6 [62.2–161.6]	123.2 [68.9–209.4]	0.394
Follow up duration (mo)	34.7 [27.7–50.9]	30.9 [14.8–44.8]	0.163
DM	9 (16.7%)	3 (13.0%)	0.690
HBV	5 (9.1%)	1 (4.3%)	0.476
HCV	8 (14.5%)	4 (17.4%)	0.752
HLA mismatches	2.0 [0.0–3.5]	0.0 [0.0–1.0]	0.279
PRA			
Class I	0.0 [0.0–15.6]	0.0 [0.0–0.0]	0.293
Class II	19.0 [0.0–58.5]	32.0 [0.0–81.7]	0.911
Induction			0.657
ATG	1 (1.8%)	0 (0.0%)	0.388
Anti-CD 25	12 (28.6%)	3 (13.0%)	
No induction	30 (71.4%)	20 (87.0%)	
Regimen			0.498
CsA based	16 (30.2%)	7 (38.9%)	
FK-506 based	37 (69.8%)	11 (61.1%)	
Drug level			
CsA (ng/ml)	101.0 [89.1–121.0]	122.0 [91.1–132.0]	0.697
FK-506 (ng/ml)	5.3 [4.2–6.2]	5.3 [3.7–6.8]	0.663
Creatinine (mg/dL)	1.8 [1.4–2.4]	1.84 [1.5–2.9]	0.635
eGFR (ml/min/1.73m^2^)	31.3 [23.3–45.8]	24.7[19.9–40.3]	0.148
Proteinuria (g/d)	0.5 [0.2–1.7]	1.5 [0.4–2.0]	0.094
Banff score			
cg	1.0 [0.0–2.0]	1.5 [1.0–3.0]	0.052
ci + ct	2.0 [2.0–2.0]	2.0 [2.0–2.5]	0.136
mm	1.0 [1.0–2.0]	1.0 [1.0–2.0]	0.959
g + ptc	3.5 [2.0–4.0]	3.0 [2.0–3.0]	0.158

*cg* transplant glomerulopathy, *ci* interstitial fibrosis, *ct* tubular atrophy, *mm* mesangial matrix increase, *g* glomerulitis, *ptc* peritubular capillary inflammation

### Survival analysis

**Fig. 1:**
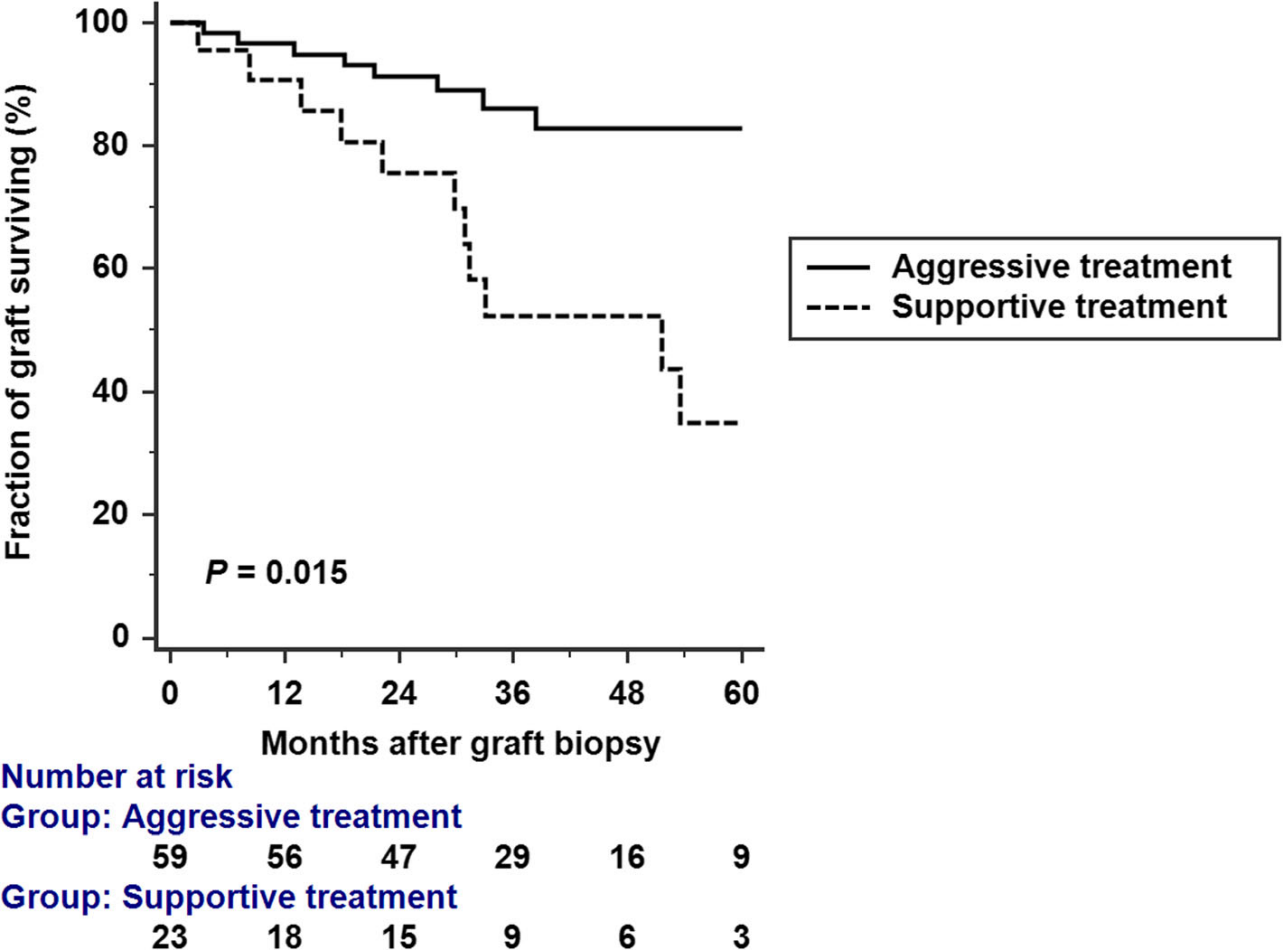
Kaplan-Meier graft survival analysis. Graft survival was constructed for aggressive treatment group and supportive treatment group. Kaplan-Meier analysis of death-censored graft survival showed a significantly worse survival in supportive treatment group (*P* = 0.015 by log-rank test)

Patients were followed for a median of 32.59 (IQR 24.01–49.89) months after the diagnosis of CAMR. A total of 22 (26.82%) patients lost their allograft, including 11/59 patients (18.64%) in group 1 and 11/23 (47.83%) patients in group 2. Median graft survival was 6.45 and 3.68 years for group 1 and group 2, respectively. Overall median graft survival was 5.6 years. Kaplan-Meier analysis of death-censored graft survival showed worse survival in group 2 (*P* = 0.015 by log-rank test) (Fig. 1).

**Fig. 2:**
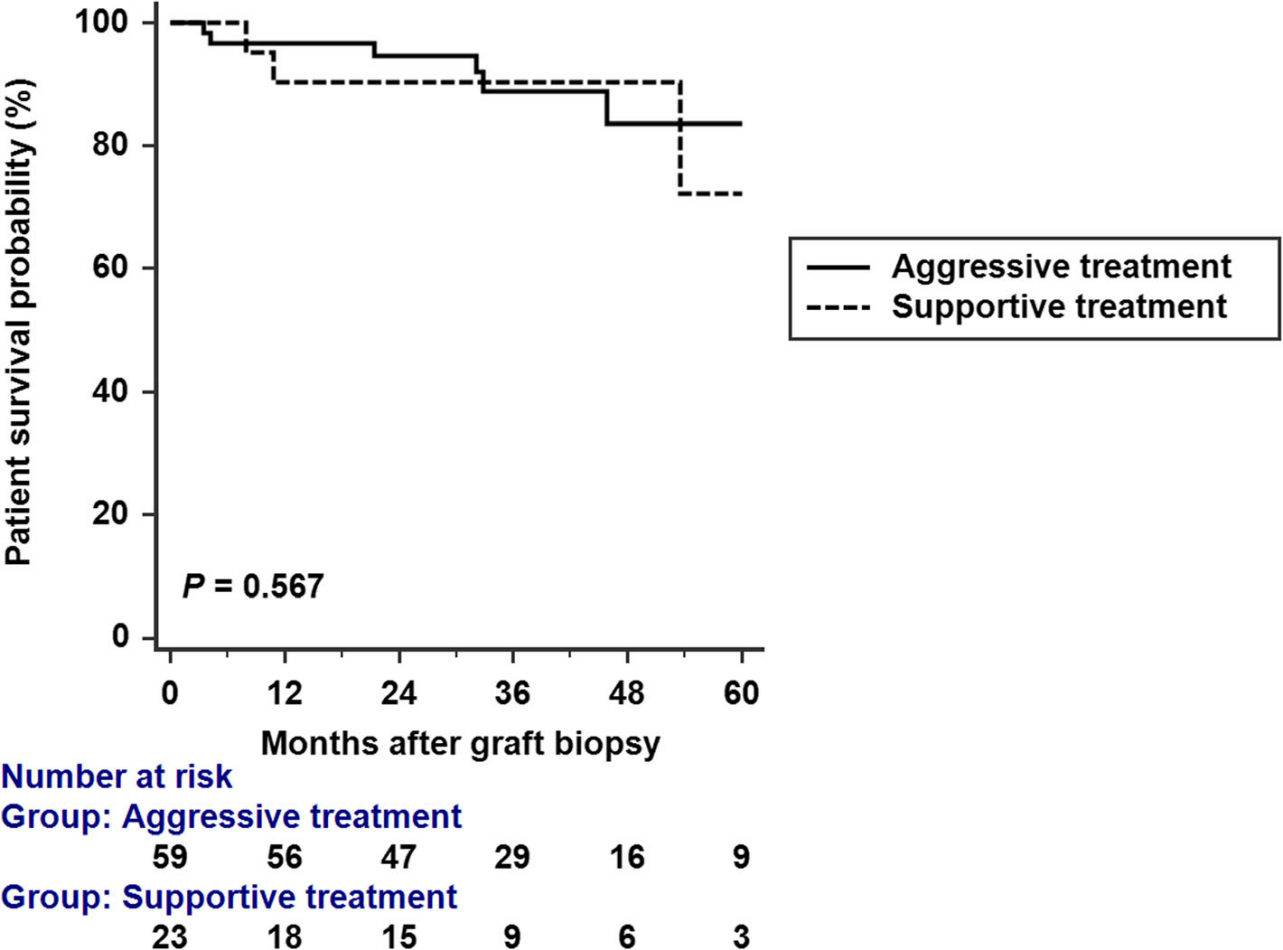
Kaplan-Meier analysis of patient survival. Study groups did not significantly differ in Kaplan-Meier patient survival (*P* = 0.567 by log-rank test)

A total of 9 (10.97%) patients died after diagnosis of CAMR, including 6/59 (10.16%) patients in group 1, and 3/23 (13.04%) in group 2. All of the mortality cases in group 1 died of sepsis. On the other hand, two of those in group 2 died of sepsis, and 1 case died of hemorrhagic shock due to hemothorax. Patient survival at the end of this study was not significantly different between these groups (*P* = 0.567 by log-rank test) (Fig. 2).

### Predictors of graft loss

By univariate analysis, the significant predictors of graft loss for CAMR were creatinine, proteinuria, PRA class II, cg ≥ 1, ci + ct ≥ 3, and supportive treatment. We constructed a multivariate regression model for graft loss analysis adjusting for proteinuria, creatinine, cg score and aggressive treatment. Supportive treatment (HR 2.86, 95%CI [1.05–7.77]) and proteinuria (HR 1.39, 95% CI [1.06–1.83])were independently associated with graft loss (Table 2).

**Table 2:** Predictors of death-censored graft loss in CAMR patients

A. Univariate analysis		
Predictor	Hazard Ratio (95% CI)	*P* Value
Age	1.02 (0.98–1.07)	0.518
Creatinine (mg/dl)	1.31 (1.12–1.52)	0.002
PRA class I	1.01 (0.99–1.02)	0.186
PRA class II	1.03 (1.01–1.04)	0.002
Proteinuria, g/d	1.37 (1.15–1.64)	0.0004
cg score ≥ 1	4.97 (1.47–16.65)	0.009
(ci + ct) ≥ 3	6.32 (2.01–19.85)	0.002
C4d score ≥ 1	1.36 (0.58–3.19)	0.476
mm score ≥ 1	1.82 (0.48–6.84)	0.374
Transplant duration (mo)	1.00 (0.99–1.01)	0.68
No treatment	2.77 (1.19–6.41)	0.017
B. Multivariable analysis ¶		
Predictor	Hazard ratio	*P* value
Supportive treatment	2.86 (1.05–7.77)	0.038
Proteinuria (g/d)	1.39 (1.06–1.83)	0.016
Creatinine (mg/dl)	1.11 (0.73–1.68)	0.621
cg score ≥ 1	3.00 (0.81–11.22)	0.102

¶The multivariate model was adjusted for the following parameters: proteinuria, creatinine, cg score, and treatment strategy

### Adverse events

**Fig. 3:**
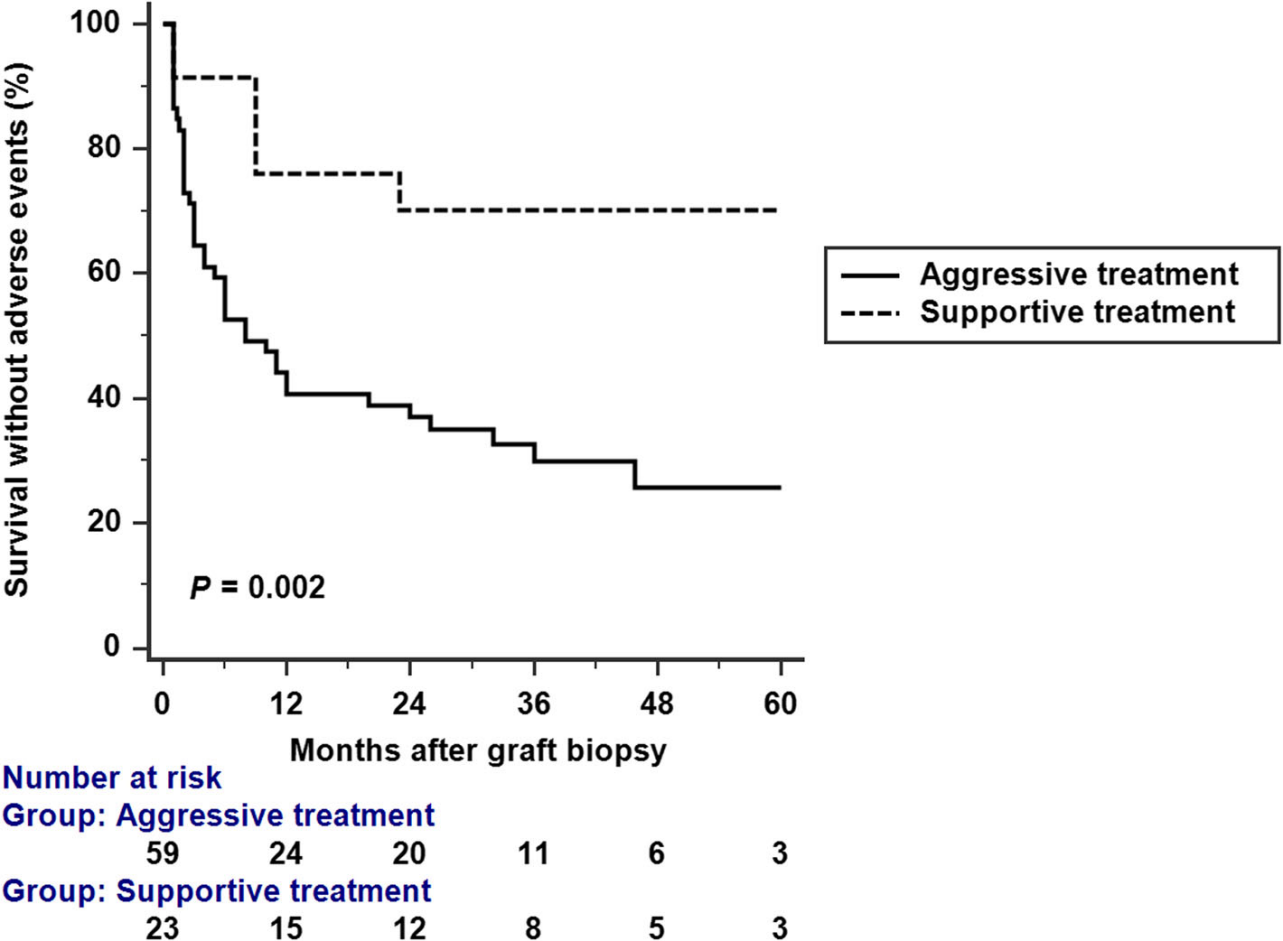
Kaplan-Meir analysis of the occurrence of major adverse events. Survival without adverse events was significantly reduced in the aggressive treatment group (*P* = 0.002 by log-rank test)

Major adverse events were demonstrated in Table 3. There was a total of 54 adverse events in group 1, compared with 7 in group 2. Mean number of adverse events per patient was higher in group 1 (*P* <  0.001). Adverse event free survival was significantly better in group 2 (*P* = 0.002 by log-rank test) (Fig. 3). The most frequent adverse events in aggressive treatment group were CMV disease, leucopenia, urinary tract infection, pneumonia, infectious diarrhea, and *Pneumocystis carinii* pneumonia (PCP). Median adverse event free survival was 6.0 (95% CI: 3–24) months in the aggressive treatment group.

**Table 3:** Major Complications. (Definition: admission, organ failure or mortality)

	Group 1, Aggressive treatment (*N* = 59)	Group 2, Supportive treatment (*N* = 23)	*P* - Value
Infection			
CMV disease	10	3	0.663
Bacterial pneumonia	9	2	0.433
PCP pneumonia	4	0	0.650
Cryptococcal pneumonia	1	0	0.505
Penicillium marneffei pneumonia	1	0	0.505
Infectious diarrhea	6	1	0.372
Urinary tract infection	8	0	0.212
Epididymitis	1	0	0.505
Cellulitis	3	0	0.889
Abscess, retroperitoneal	1	0	0.505
Abscess, lung	1	0	0.505
Peritonitis	1	0	0.505
Fungemia	1	0	0.505
Leukopenia	8	1	0.231
Mortality¶	6	3	0.708
Total number of AEs	54	7	
Mean number AEs per patient	1.0 [0.0–2.2]	0.0 [0.0–0.7]	< 0.001

¶All the mortality cases in the aggressive treatment group died of sepsis, whereas two of those in no treatment group died of sepsis, another case died of hemorrhagic shock. *PCP Pneumocystis carinii* pneumonia. *AE* adverse events

### Subgroup analysis

**Fig. 4:**
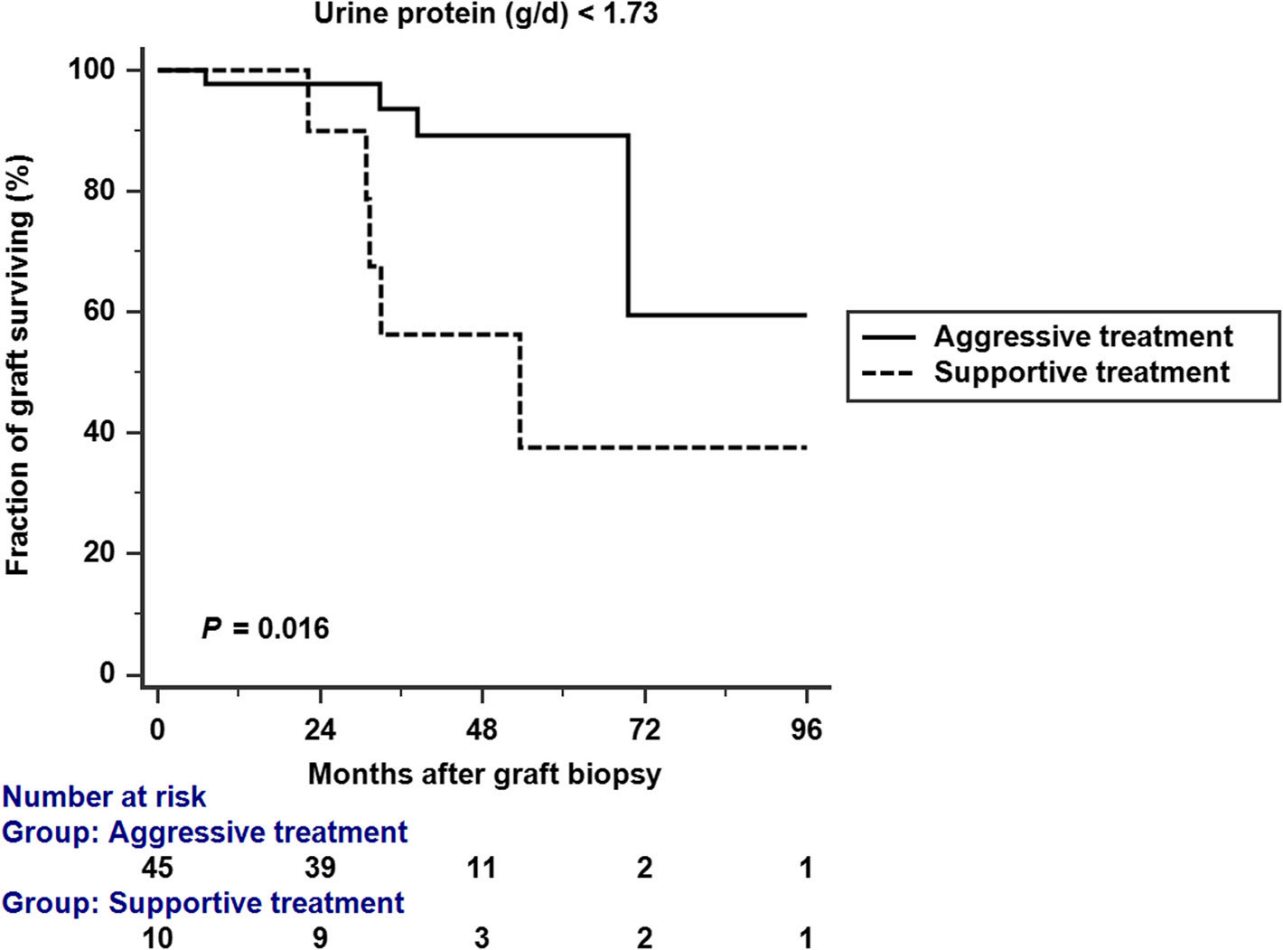
Kaplan-Meir analysis of graft survival in patients with proteinuria < 1.73 g/d. Aggressive treatment was associated with better graft survival. (*p* = 0.016 by log rank analysis)

**Fig. 5:**
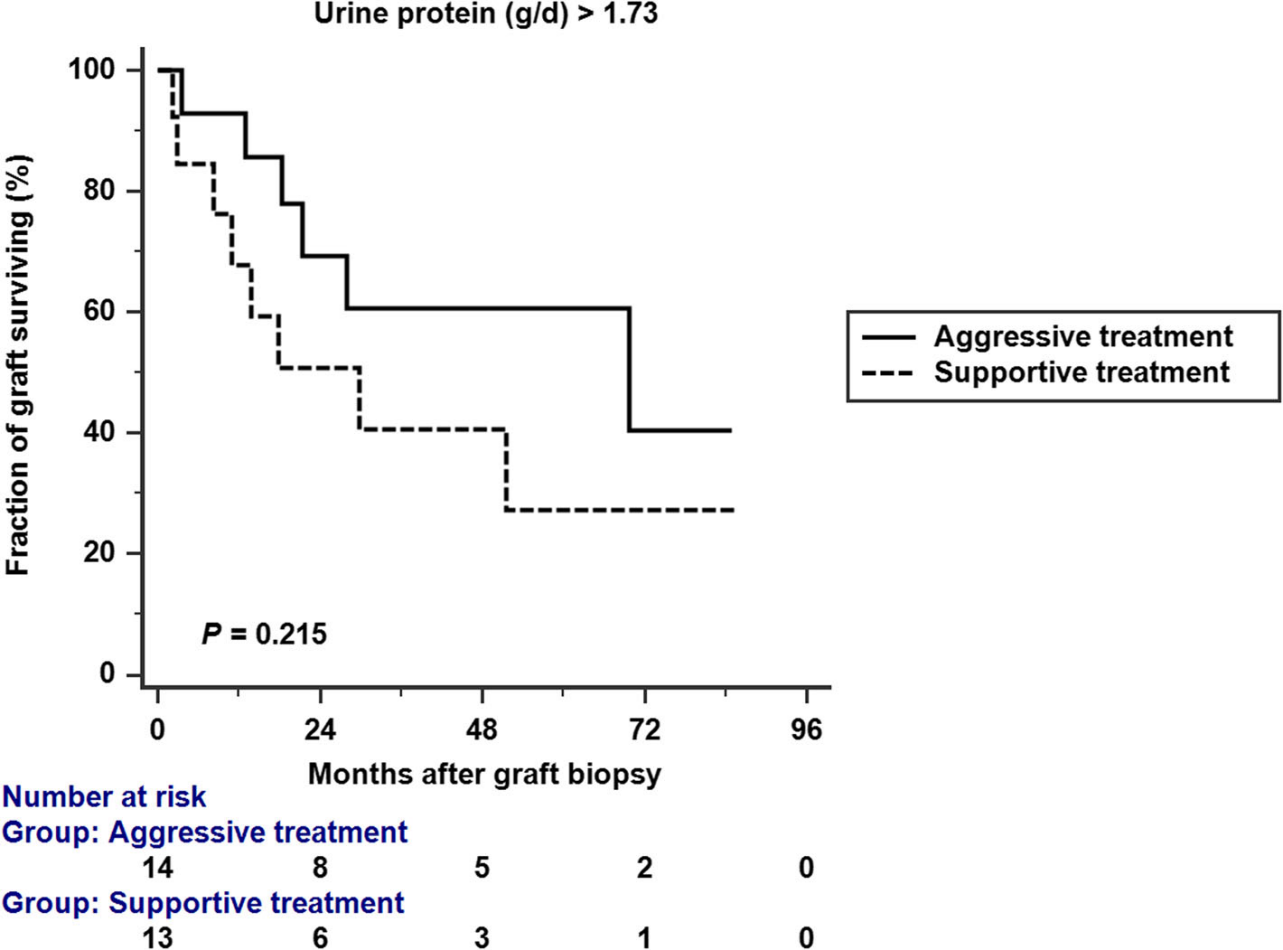
Kaplan-Meir analysis of graft survival in patients with proteinuria ≥1.73 g/d. Aggressive treatment was not significantly associated with better graft survival. (*p* = 0.215 by log rank analysis)

We conducted a Kaplan-Meier analysis of graft survival in patients with proteinuria < 1.73 g/d and ≥ 1.73 g/d. Aggressive treatment resulted in better graft survival in patients with proteinuria < 1.73 g/d (*p* = 0.016 by log rank analysis), but not in patients with proteinuria ≥1.73 g/d (*p* = 0.215 by log rank analysis) (Figs. 4; 5). In the subgroup analysis which included patients with proteinuria < 1.73 g/d (Table 4), there was no significant difference between aggressive treatment and supportive treatment group in terms of proteinuria, creatinine, and Banff scores.

**Table 4:** Subgroup analysis of patients with daily urine protein < 1.73 g

	Group 1, Aggressive treatment (*N* = 45)	Group2, Supportive treatment (*N* = 10)	*P* value
Age at biopsy	50.2 [40.5–58.7]	50.9 [46.9–63.9]	0.294
Creatinine (mg/dL)	1.8 [1.4–2.4]	1.7 [1.5–2.3]	0.768
eGFR (ml/min/1.73m^2^)	33.2 [23.8–48.1]	30.9 [22.5–46.7]	0.751
Proteinuria (g/d)	0.3 [0.2–0.6]	0.5 [0.3–0.8]	0.111
Regimen			
CsA based	10 (22.2%)	3 (30.0%)	
FK-506 based	35 (77.8%)	7 (70.0%)	
Drug level			
CsA (ng/ml)	90.6 [86.4–115.0]	88.3 [84.2–96.5]	0.637
FK-506 (ng/ml)	5.3 [4.3–6.2]	5.2 [3.9–6.8]	0.959
Banff score			
cg	1.0 [0.0–1.0]	1.0 [0.0–1.0]	0.683
ci + ct	2.0 [0.0–2.0]	2.0 [2.0–2.0]	0.269
mm	1.0 [1.0–2.0]	1.0 [1.0–2.0]	0.943
g + ptc	3.0 [2.0–4.0]	3.0 [2.0–3.0]	0.445

*cg* transplant glomerulopathy, *ci* interstitial fibrosis, *ct* tubular atrophy, *mm* mesangial matrix increase, *g* glomerulitis, *ptc* peritubular capillary inflammation

## Discussion

We found that aggressive treatment for CAMR patients was associated with better graft survival. However, the aggressive treatment group also had higher incidence of adverse events and a reduced adverse event free survival. The factors independently associated with graft loss were proteinuria and supportive treatment.

Currently, there are no approved treatments for CAMR. Billing et al. reported a study on IVIG and rituximab treatment in 20 paediatric renal transplant recipients with CAMR. They reported that IVIG and rituximab significantly reduced or stabilized the progressive loss of transplant function [3, 10] However, the subgroup with transplant glomerulopathy (TG) was associated with a poorer response. Another study conducted by Bachelet et al. showed IVIG with rituximab treatment for severe TG in CAMR did not change the natural history of TG [4]. Recently, a multicenter, prospective, randomized double-blind clinical trial for evaluation the efficacy and safety of IVIG with rituximab also revealed no difference between the treatment and placebo groups in eGFR decline, increase of proteinuria, and MFI of the immunodominant DSA. The author considered the presence of TG as an inclusion criteria (mean cg score in the treatment group: 2.3 ± 0.8), and this may be the reason of a poor response in this study [11]. In fact, there was evidence that the combination of IVIG and rituximab appeared to be beneficial in patients with high levels of microvascular injury, for example biopsies with g ≥ 2 and/or (g + ptc) ≥ 4 [12]. On the contrary, patients with low microvascular injury scores appeared less likely to benefit from antihumoral therapy.

Bortezomib has also been evaluated in patients with CAMR. Clinical experience of bortezomib in transplantation showed variable results among patients with different disease states and populations. Recently, a randomized, placebo-controlled trial demonstrated that two cycles of bortezomib had no significant benefit for late onset DSA-positive ABMR in graft survival and DSA reduction [5]. Advanced tissue injury and higher proportion of preformed DSAs in this study might be a possible explanation. Moreover, HLA antibodies produced by long-lived plasma cells (LLPCs) are more refractory to proteasome inhibitor therapy. LLPC resistance and immunologic compensatory mechanisms may also play a role in treatment failure [13].

In our study, CAMR was diagnosed at a relatively early stage (median cg score: 1.0, ci + ct: 2.0) compared to previous studies (mean cg score: 2.0; ci + ct: 3.5 in a recent clinical trial [11] and mean cg score: 2.2; ci + ct score: 2.8 in a previous retrospective study [4]). Furthermore, the microvascular injury was prominent (median [g + ptc] score: 3.5). The above characteristics made our patients more likely to respond to antihumoral therapy. The graft survival was significantly better in the aggressive treatment group compared to the supportive treatment group. Supportive treatment was a predictor of graft loss in the univariate analysis (HR 2.77, 95% CI [1.19–6.41], *P* = 0.017). After adjustment of proteinuria, creatinine, and cg score, supportive treatment was still an independent risk factor of graft loss (HR 2.86, 95%CI [1.05–7.77], *P* = 0.038). **A** subgroup analysis revealed that aggressive treatment for CAMR resulted in better graft survival in patients with proteinuria < 1.73 g/d but not in patients with proteinuria ≥1.73 g/d. Our study highlight the importance of aggressive treatment in CAMR at an earlier stage and with a higher degree of microvascular injury.

Rituximab, IVIG, and bortezomib treatment are not reimbursed by Taiwan's National Health Insurance program and should be self-paid. Therefore, some patients received methylprednisolone pulse therapy with plasmapheresis only. These patients had graft survival between aggressive treatment and supportive treatment group (Additional file 1: Figure S1). Previously, Redfield et al. had reported a retrospective study for outcome of CAMR [14]. The author divided their patients into three groups: steroid/IVIG with rituximab or antithymocyte globulin, steroid/IVIG alone or in combination, and no treatment. The most aggressive treatment group had the best graft survival, which was in line with our study. However, the graft biopsies of CAMR in Redfield’s series had relatively advanced disease (median cg of 2 and proteinuria > 1 g). Therefore, the graft survival in our study was better (overall median graft survival 5.4 years vs. 1.9 years).

Following aggressive treatment of CAMR, adverse event is an important issue. The most frequently prescribed antihumoral agent for our patients was rituximab, followed by IVIG, bortezomib, and ATG. In a retrospective study published by Kamar et al., 9.1% of kidney transplant patients died of infectious diseases after rituximab treatment [15]. This result was similar to that in our aggressive treatment group (10.16%). The most common adverse events in our patients were CMV disease, urinary tract infection, bacterial / PCP pneumonia, and infectious diarrhea.

CMV infection was reported to be associated with Rituximab, ATG, and bortezomib treatment in renal transplant and myeloma patients [16–18]. ABO-incompatible kidney transplant recipients who received rituximab had higher incidence of CMV disease [16]. Studies revealed an increased frequency of CMV disease associated with ATG treatment, probably due to the release of TNF-α after ATG administration, which may stimulate cellular nuclear factor кB and viral replication via binding to the promoter region of the CMV immediate-early antigen gene [17]. Furthermore, several studies indicated that bortezomib treatment is associated with higher risk of viral infection, including CMV [18–20]. Basler M et al. demonstrated reduced cytotoxic T cell response and impaired viral clearance in bortezomib treated mice [19].

PCP is a major cause of morbidity and mortality in patients receiving immunosuppressant therapies. Previous CMV infection, acute graft rejection and intensity of immunosuppressive therapy had been reported as risk factors for PCP in kidney transplant recipients [21].

Among the mortality cases in our aggressive treatment group, 3 of 6 died of CMV disease, including 1 CMV pneumonia and 2 CMV colitis. Adequate valganciclovir prophylaxis may have reduced the mortality rate by 50% in our patients who received aggressive treatment.

Kamar et al. demonstrated that the median duration between last rituximab and first infection episode in kidney transplant recipients was about 5 months [15], which was in accordance with our study (6 months). Our previous policy about prophylactic antibiotics (valganciclovir and trimethoprim-sulfamethoxazole) was to give these 2 agents for just 1 month after aggressive treatment. In this regards, it is reasonable to recommend that valganciclovir and trimethoprim-sulfamethoxazole prophylaxis be given for at least 5~6 months after aggressive anti-rejection therapy.

Despite the significantly higher rate of adverse events in the aggressive treatment group, there was no significant difference in patient survival (Fig. 2), implying that the patients still could have a reasonable chance of survival if these complications can be treated judiciously.

There are limitations in our study. Firstly, there were no rules for treatment of CAMR in our cohort, and the need for treatment was determined by each clinical physician. Second, DSA was not performed for every recipients in our hospital, because Luminex® technology for HLA antibody detection in organ transplant is expensive in Taiwan. On the other hand, PRA is reimbursed by Taiwan's National Health Insurance system and thus we performed PRA for our kidney transplant recipients every year.

## Conclusion

In conclusion, aggressive treatment for CAMR before advanced tissue injury is still associated with better graft outcome in our series. However, higher incidence of adverse events cannot be overlooked. To mitigate potential life-threatening infections, longer duration of PCP and CMV prophylaxis should be considered after aggressive treatment for rejection.

## Additional file

**Additional file 1.** Kaplan-Meier graft survival analysis. Graft survival was analyzed for three different treatment strategies: aggressive treatment (exclude those who received MP pulse with DFPP only), MP pulse with DFPP, and supportive treatment.

## Data Availability

The individual patient-level data was not made publically available due to containing potentially identifying patient data; however, the study data may be made available from the authors upon reasonable request.

## Abbreviations

ATG: Antithymogycte globulin
CAMR: Chronic antibody-mediated rejection
DFPP: Double filtration plasmapheresis
DSA: Donor-specific antibody
eGFR: Estimated glomerular filtration rate
GN: Glomerulonephritis
IVIG: Intravenous immunoglobulin
MP: Methylprednisolone
PCP: *Pneumocystis carinii* pneumonia
PRA: Panel-reactive antibody
