# Supplementary figures and images for: Treatment of chronic active antibody-mediated rejection in renal transplant recipients – a single center retrospective study
Source: BMC Nephrol. 2020 Jan 6;21:6. doi: 10.1186/s12882-019-1672-8 (PMC6945538; doi:10.1186/s12882-019-1672-8)

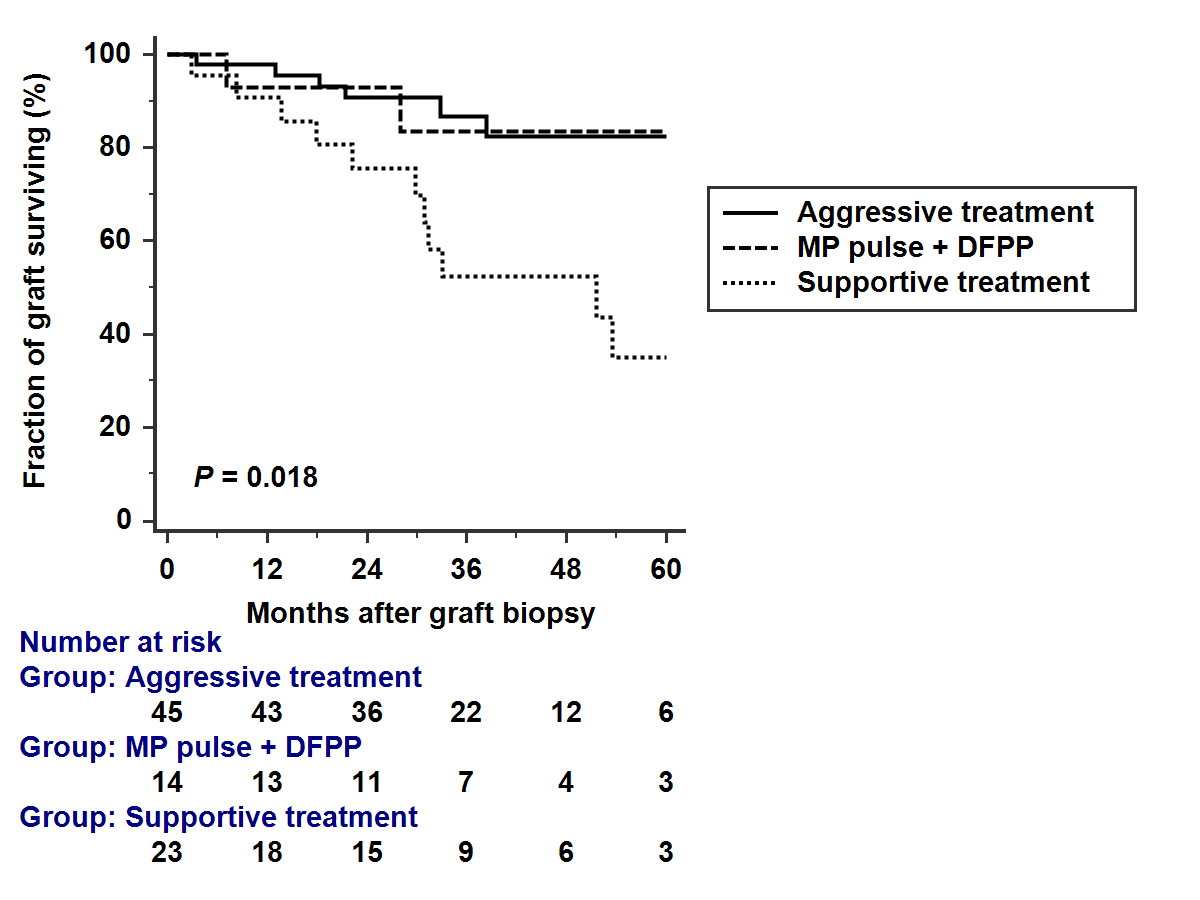

Supplement: Supplementary file 1 — Additional file 1. Kaplan-Meier graft survival analysis. Graft survival was analyzed for three different treatment strategies: aggressive treatment (exclude those who received MP pulse with DFPP only), MP pulse with DFPP, and supportive treatment. [file 12882_2019_1672_MOESM1_ESM.tif]
